# Comparative **e**valuation of SBS and RASS for **s**edation **a**ssessment in **m**echanically **v**entilated **c**hildren

**DOI:** 10.3389/fped.2026.1829799

**Published:** 2026-05-25

**Authors:** Dan Li, Shan He, Qi Li, Jie Shen, Ping Tang

**Affiliations:** Department of Critical Care Medicine, Children’s Hospital of Chongqing Medical University, National Clinical Research Center for Children and Adolescents’ Health and Diseases, Ministry of Education Key Laboratory of Child Development and Disorders, Chongqing Key Laboratory of Child Neurodevelopment and Cognitive Disorders, Chongqing, China

**Keywords:** mechanical ventilation, pediatric intensive care unit, richmond agitation-sedation scale, sedation, State Behavioral Scale

## Abstract

**Background:**

In mechanically ventilated children in the PICU, assessment of analgesia, sedation, and delirium often requires multiple instruments, which increases workflow complexity and nursing burden.

**Objective:**

To evaluate the association and clinical agreement between the State Behavioral Scale (SBS) and the Richmond Agitation-Sedation Scale (RASS) in mechanically ventilated children in the PICU.

**Methods:**

This retrospective study included 1,025 mechanically ventilated children admitted to a tertiary PICU between May 2024 and October 2025. SBS and RASS scores were recorded simultaneously at predefined time points, resulting in 13,736 paired observations. Patient-level clustering caused by repeated measurements was addressed using cluster-robust standard errors and generalized estimating equations (GEE).

**Results:**

Spearman rank correlation analysis with patient-level cluster-robust standard errors showed a strong positive association between RASS and SBS scores (*r* = 0.857, 95% CI: 0.841–0.873, *p* < 0.001). Linear weighted kappa demonstrated good agreement (weighted kappa = 0.821, 95% CI: 0.802–0.840), and the GEE-adjusted robust coefficient remained stable (0.849, 95% CI: 0.833–0.865, *p* < 0.001). In a nurse survey, 64.7% reported that RASS was more convenient and faster to use, and 54.9% considered it more reflective of clinical status.

**Conclusions:**

RASS showed good clinical agreement with SBS in mechanically ventilated children and may serve as a practical and efficient alternative for sedation assessment in the PICU, provided that standardized training and local implementation procedures are ensured.

## Introduction

The pediatric intensive care unit (PICU) provides continuous, high-acuity care for critically ill children. Critically ill children in the PICU are exposed to substantial physiological and psychological stress due to severe illness, invasive procedures, mechanical ventilation, and an unfamiliar environment. Although mechanical ventilation is essential for stabilizing vital signs, it can also cause pain, anxiety, agitation, and discomfort. Sedation is therefore an essential component of pediatric critical care management (1). Sedation depth must be carefully titrated: undersedation may increase stress and lead to adverse events such as accidental extubation, whereas oversedation can prolong mechanical ventilation and PICU stay and increase the risk of complications, including iatrogenic withdrawal syndrome and delirium (1). Accurate bedside assessment is therefore essential for individualized sedation adjustment (2, 3). Current practice increasingly favors light, goal-directed sedation to maintain comfort and safety while supporting arousal, early mobilization, and recovery (4, 5). This approach depends on reliable tools that can monitor sedation depth consistently over time (6).

Several sedation scales are used in the PICU, including the Ramsay Sedation Scale, the COMFORT scale, the RASS, and the Sedation-Agitation Scale (1, 7–10). The RASS is recommended by the 2022 Society of Critical Care Medicine guidelines and by multiple adult ICU studies because it is simple to administer and has strong validity (6, 11). For mechanically ventilated children, the State Behavioral Scale (SBS) remains the recommended primary sedation assessment tool (12). Inappropriate sedative exposure is also associated with pediatric delirium (11, 13, 14). Accordingly, delirium assessment often combines the Cornell Assessment of Pediatric Delirium with a preceding RASS assessment of arousal and sedation status (8, 12, 15–17). Although RASS was developed for adult ICUs (18), pediatric studies have reported good reliability and validity (19–21); however, the evidence base remains limited by relatively small or single-center samples, and guideline recommendations for pediatric use remain cautious (12, 22).

In routine PICU practice, nurses may need to apply several assessment tools during the same shift. This increases workload and may reduce the efficiency of sedation and delirium screening (2). In many units, SBS is used for sedation assessment, whereas a RASS-based workflow is used before delirium assessment. However, whether RASS can provide clinically comparable sedation assessment to SBS in mechanically ventilated children remains unclear.

## 1 Subjects and Methods

### 1.1 Subjects

This study was approved by the Medical Ethics Committee of Children's Hospital of Chongqing Medical University, with approval number: (2026) Ethics Approve No. (21), and approval date: February 8, 2026. This retrospective observational study included mechanically ventilated children admitted to the PICU of Children's Hospital of Chongqing Medical University between May 2024 and October 2025. This study period was selected because complete routinely collected electronic sedation assessment records were available for this interval. Inclusion criteria were: (1) age 29 days to 18 years; (2) continuous sedation for at least 24 h; (3) no severe neurological injury and no use of neuromuscular blocking agents; and (4) mechanical ventilation for at least 24 h in the PICU. Comatose children (GCS ≦ 8), neuromuscular blocking agent use, or missing key assessment data were excluded.

### 1.2 Methods

All nurses had received standardized training in SBS and RASS assessment before data collection. Patients were managed according to the standard PICU sedation protocol. Midazolam was administered by continuous intravenous infusion at a maintenance dose of 1–4 μg/kg/min, Combined fentanyl (1–4 μg/kg/h) and sufentanil (0.04–0.15 μg/kg/h) for analgesia, with dose adjustments not exceeding 10% per adjustment. For pediatric patients with inadequate sedation and significant agitation, propofol (0.5–1 mg/kg) was administered via intravenous bolus injection or fentanyl (1–2 μg/kg) was supplemented on an ad-hoc basis according to clinical condition. In some children with a predisposition to convulsions, a single loading dose of phenobarbital (5–10 mg/kg) was given. Prior to ventilator weaning and extubation, the sedatives were gradually tapered to a light sedation level without abrupt discontinuation. For those on continuous medication for 7 days or longer, the dose was reduced by 20%–30% daily. Sedation depth was individualized according to disease severity, ventilation mode, and repeated bedside assessment results. Meanwhile, a questionnaire survey was conducted among 51 nursing staff participants, covering educational background, working years, and the ease of use and clinical conformity of the SBS and RASS scales (Attached Table 2). This survey adopted convenience sampling without reliability and validity testing, and only descriptive analysis was performed.

#### 1.2.1 Study tools

Baseline clinical data included age, sex, diagnosis, sedative dose and duration, ventilation mode and duration, PICU length of stay, and vital signs. Sedation was assessed using both SBS and RASS. The SBS is a validated tool designed for sedation assessment in mechanically ventilated children and evaluates sedation and agitation through observable behavioral responses (9, 12, 23). Scores range from −3 (unresponsive) to +2 (agitated). The RASS is a validated arousal and sedation scale widely used in adult ICUs and increasingly applied in pediatric settings; it is also incorporated into delirium assessment workflows such as CAPD screening (8, 24). RASS scores range from −5 to +4, with lower scores indicating deeper sedation and higher scores indicating agitation. The nurse survey evaluated perceived speed, convenience, and clinical usefulness of the two scales.

#### 1.2.2 Sample size

Because this was a retrospective analysis of routinely collected clinical data, no formal *a priori* sample size calculation was performed. Instead, all eligible patients within the predefined data-availability period were included. A total of 1,025 children contributed 13,736 paired SBS-RASS assessments.

#### 1.2.3 Data collection

Patients meeting the inclusion criteria were included. SBS and RASS were recorded simultaneously at four predefined time points each day (09:00, 15:00, 21:00, and 03:00). Assessments were performed when no procedures or acute interventions were taking place. Assessments began 24 h after sedation initiation and continued until extubation. The primary analysis used paired SBS and RASS measurements obtained at the same time points; patient identification was retained as the clustering variable for analyses addressing repeated measurements. Assessments followed a standardized two-step approach: observation followed by stimulation when needed.

### 1.3 Statistical analysis

Statistical analyses were performed using Python (version 3.0). Continuous variables are presented as mean ± standard deviation or median and interquartile range, as appropriate, and categorical variables are presented as frequencies and percentages. Because multiple paired observations were obtained from the same patient, patient ID was used as the clustering unit. The association between RASS and SBS scores was evaluated using Spearman rank correlation, with 95% confidence intervals estimated using patient-level cluster-robust standard errors. Agreement between the two ordinal sedation scales was quantified using the linear weighted kappa coefficient with 95% confidence intervals. To evaluate the robustness of the agreement result after accounting for repeated measurements, a generalized estimating equation (GEE) model with patient-level clustering was used as a sensitivity analysis. The model was adjusted for age, duration of mechanical ventilation, and PRISM-III score. Subgroup analyses were conducted by age, duration of mechanical ventilation, sedation level, and PRISM-III illness severity. Clinical agreement was interpreted based on weighted kappa, predefined category mapping, and GEE-adjusted estimates. A two-sided *p* value <0.05 was considered statistically significant.

## 2 Results

### 2.1 General characteristics

A total of 1,850 children were initially screened. Of these, 458 were excluded because of neurological injury or use of neuromuscular blocking agents, and 367 were excluded because of missing data. The final analysis included 1,025 children and 13,736 paired SBS-RASS assessments. Given the wide and skewed age distribution, patients were categorized into four age groups: ≤2 years (51.0%), 3–5 years (14.6%), 6–15 years (32.2%), and >15 years (2.2%) (Table 1).

**Table 1:** General information of patients (*N* = 1,025).

Clinical Characteristics	Group	Cases	Percentage
Gender	Male	606	59.12
	Female	419	40.88
Age	≤2 years	523	51.02
	3–5 years	150	14.63
	6–15 years	330	32.2
	>15 years	22	2.15
Admission Diagnosis	Respiratory diseases	245	23.8
	Circulatory diseases	196	19.12
	Neurological diseases	258	25.17
	Other diseases	327	31.91
ICU hospitalization Duration	1–3 days	365	35.61
	4–6 days	186	18.15
	7–9 days	186	18.15
	≥10 days	288	28.09
Mechanical Ventilation Duration	1–3 days	567	55.32
	4–6 days	183	17.85
	7–9 days	130	12.68
	≥10 days	145	14.15
Continuous Sedative and Analgesic Infusion Duration	1–3 days	354	34.54
	4–6 days	267	26.04
	7–9 days	156	15.22
	≥10 days	248	24.2

### 2.2 Sedation assessment results

#### 2.2.1 Distribution of sedation levels

**Figure 1:**
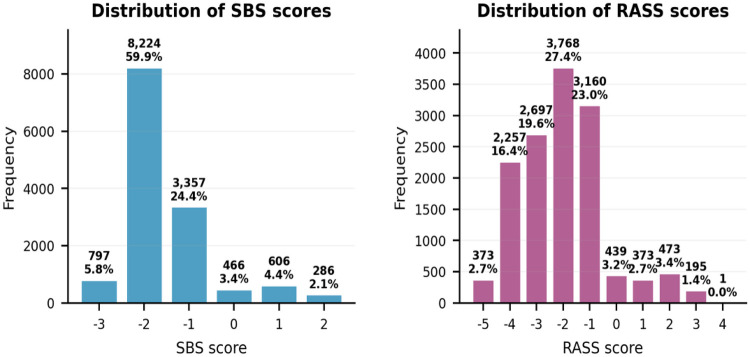
Distribution of SBS and RASS scores. Bars show counts and percentages among 13,736 paired assessments.

Most paired assessments were within the target sedation range, with smaller proportions classified as deep sedation or inadequate sedation (Figure 1).

#### 2.2.2 Correlation between SBS and RASS

**Figure 2:**
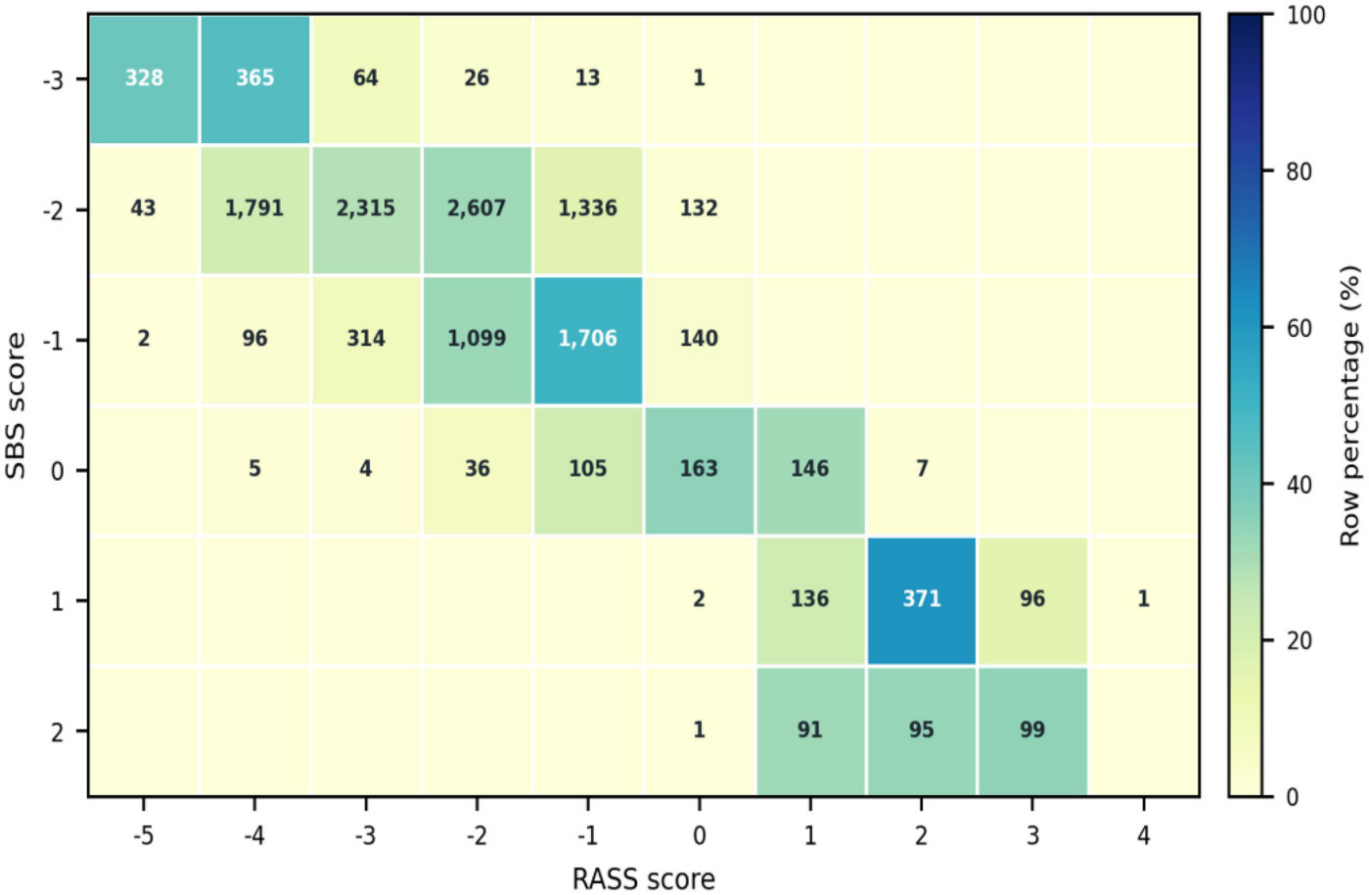
Score-level heatmap of paired SBS and RASS assessments. Cells show paired assessment counts; color intensity represents row percentage within each SBS score level.

In total, 13,736 paired assessments were included in the analysis. Spearman rank correlation analysis showed a strong positive association between RASS and SBS scores. After correction with patient-level cluster-robust standard errors to account for repeated measurements within the same child, the correlation remained strong and statistically significant (*r* = 0.857, 95% CI: 0.841–0.873, *p* < 0.001). The score-level distribution of paired SBS and RASS assessments is shown in Figure 2.

#### 2.2.3 Agreement analysis

Consistency analysis was performed based on the clinical correspondence between the two ordered sedation scales. The results showed that SBS and RASS exhibited good consistency. The linearly weighted Kappa coefficient was 0.821 (95% CI: 0.802–0.840), exceeding the commonly used threshold of 0.80 for strong consistency.

To further control for confounding factors, multivariable generalized estimating equations (GEE) were used to adjust for age, duration of mechanical ventilation, and PRISM-III score. After adjustment, RASS and SBS scores maintained excellent agreement, with a robust weighted kappa of 0.817 (95% CI: 0.798–0.836, *P* < 0.001). In the GEE linear regression model with SBS as the dependent variable and RASS as the independent variable, the regression coefficient for RASS was 0.823 (95% CI: 0.805–0.841, *P* < 0.001). Age, ventilation duration, and PRISM-III score had no statistically significant confounding effects (all *P* > 0.05), confirming the robustness of the main findings. Subgroup analyses are presented in Table 2.

**Table 2:** Subgroup analyses of agreement between RASS and SBS using GEE-adjusted weighted kappa.

Stratification factor	Subgroup	GEE-adjusted weighted Kappa	95% CI	*P* value
Age group	≤2 years	0.809	0.788–0.830	<0.001
	3–5 years	0.835	0.810–0.860	<0.001
	6–15 years	0.826	0.805–0.847	<0.001
	>15 years	0.812	0.768–0.856	<0.001
Mechanical ventilation duration	1–3 days	0.823	0.805–0.841	<0.001
	4–6 days	0.814	0.789–0.839	<0.001
	7–9 days	0.808	0.779–0.837	<0.001
	≥10 days	0.803	0.776–0.830	<0.001
Sedation level	Deep sedation	0.782	0.745–0.819	<0.001
	Target sedation	0.837	0.821–0.853	<0.001
	Under-sedation	0.829	0.802–0.856	<0.001
PRISM-III severity	Mild	0.835	0.813–0.857	<0.001
	Moderate	0.818	0.797–0.839	<0.001
	Severe	0.801	0.773–0.829	<0.001

**Figure 3:**
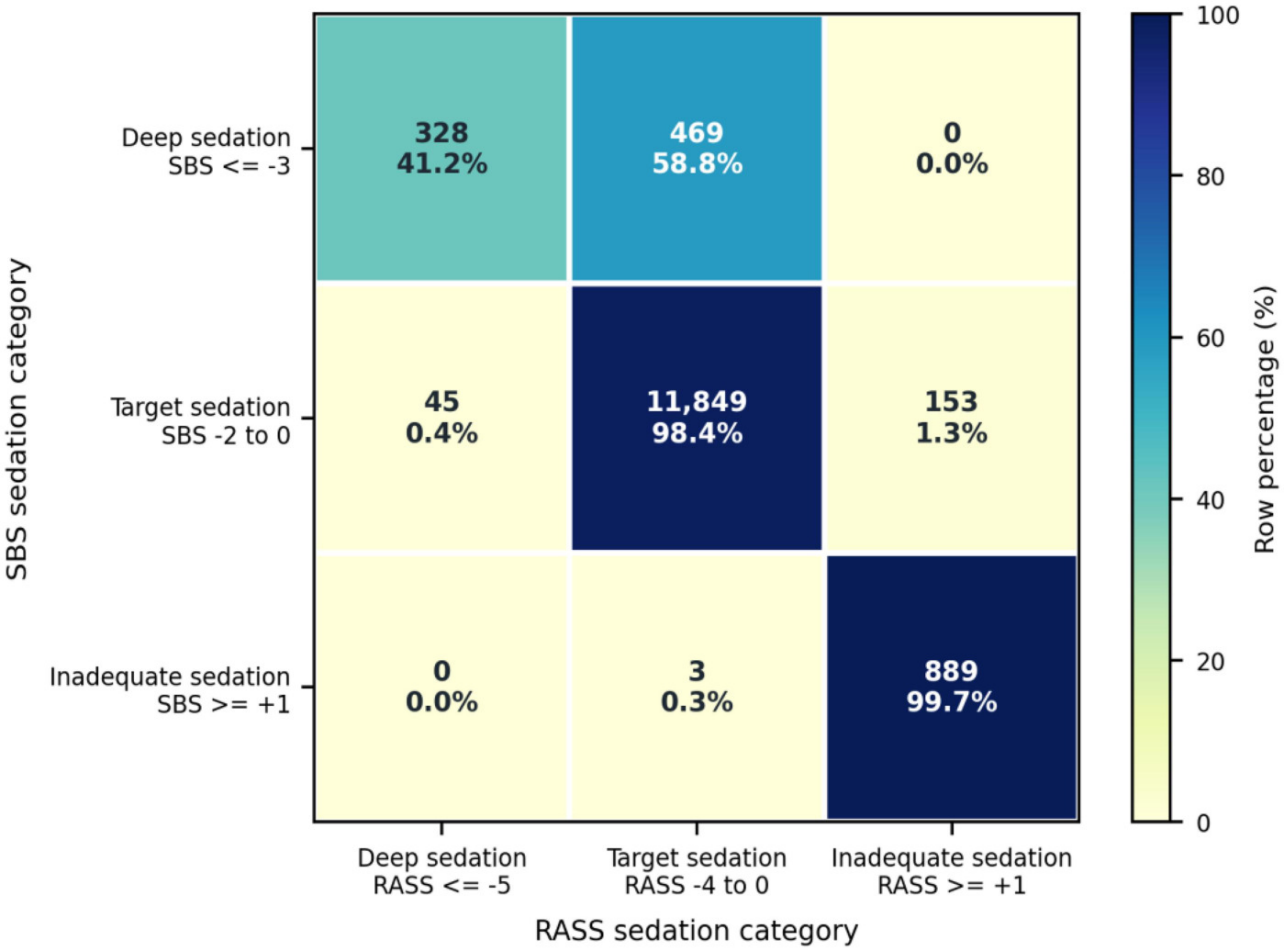
Clinical-category agreement matrix between SBS and RASS.

In the GEE sensitivity analysis accounting for patient-level clustering, the robust coefficient was 0.849 (95% CI: 0.833–0.865, *P* < 0.001), indicating that the agreement result was stable after adjustment for repeated measurements. The clinical-category agreement matrix is presented in Figure 3. The paired assessments clustered mainly within clinically corresponding sedation ranges, while the deepest sedation category showed the greatest discrepancy between SBS and RASS, suggesting that the two scales may differ most at the deepest arousal levels. Table 3 summarizes the mean scores and distribution of paired assessments across deep, target, and inadequate sedation zones for both scales.

**Table 3:** Average scores and proportions by sedation zone for SBS and RASS (*N* = 13,736 paired assessments).

Assessment tool	Deep sedation		Target sedation		Inadequate sedation	
	Score, mean ± SD	*n* (%)	Score, mean ± SD	*n* (%)	Score, mean ± SD	*n* (%)
SBS	−3.00 ± 0.00	797 (5.80)	−1.64 ± 0.55	12,047 (87.70)	1.32 ± 0.47	892 (6.49)
RASS	−5.00 ± 0.00	373 (2.72)	−2.26 ± 1.13	12,321 (89.70)	1.83 ± 0.72	1,042 (7.59)

Cells show paired assessment counts and row percentages within each SBS sedation category.

## 3 Discussion

This study systematically evaluated the agreement and clinical applicability of the State Behavioral Scale (SBS) and the Richmond Agitation-Sedation Scale (RASS) in mechanically ventilated children in the PICU. The findings demonstrate a strong correlation and good agreement between the two instruments across clinically relevant sedation levels, indicating that both scales provide comparable assessments of sedation depth in most clinical scenarios. These results are consistent with previous pediatric studies reporting good validity and reliability of RASS in critically ill children (19–21, 24), supporting its potential applicability beyond adult ICU settings.

A key finding of this study is the high level of agreement between SBS and RASS within clinically meaningful sedation categories. In particular, the concordance was most pronounced in the target sedation and inadequate sedation ranges, which are highly relevant for routine clinical management. This suggests that both instruments can reliably reflect sedation status under typical PICU conditions, especially when sedation is titrated toward predefined goals. From a clinical perspective, this level of agreement is important because it implies that RASS can provide sedation information that is functionally equivalent to SBS in guiding bedside decision-making. Given that current guidelines emphasize goal-directed, relatively light sedation in critically ill children (12, 22, 25), the ability of RASS to accurately capture target sedation states further supports its clinical utility.

To further confirm the reliability of our results, we performed multi-variable GEE analyses adjusted for age, duration of mechanical ventilation, and PRISM-III illness severity. Subgroup analyses were also conducted across different age groups, ventilation durations, sedation levels, and illness severity strata. The excellent agreement between RASS and SBS remained stable after adjustment and stratification, indicating that our main findings were robust and not significantly affected by these clinical factors.

Despite the overall agreement, discrepancies between the two scales were observed, particularly in the deepest levels of sedation. This finding is clinically relevant and may be explained by inherent differences in scale design. RASS includes more granular levels at the deep sedation end, allowing for finer discrimination of arousal responses, whereas SBS categorization is relatively broader. As a result, subtle variations in patient responsiveness may be classified differently between the two instruments. In addition, differences in behavioral descriptors and bedside assessment practices may contribute to variability in deep sedation scoring. Similar inconsistencies in extreme sedation ranges have been reported in previous studies evaluating sedation assessment tools (6). These findings suggest that, while the two scales are broadly interchangeable in most situations, caution is warranted when assessing patients at very deep levels of sedation, where scale-specific characteristics may influence interpretation.

From a practical standpoint, the potential of RASS to serve as an alternative to SBS has important implications for clinical workflow. In many PICU settings, nurses are required to use multiple assessment tools for sedation and delirium evaluation, which increases workload and may reduce efficiency. In this study, nurse feedback indicated that RASS was perceived as faster, more convenient, and more reflective of clinical status. These findings are consistent with previous research suggesting that simpler and more intuitive tools are more readily adopted in routine ICU practice (6). Importantly, RASS is already widely used as part of delirium assessment workflows, such as preceding CAPD evaluation, which creates an opportunity to integrate sedation and delirium monitoring into a unified framework (14, 17). From this perspective, the use of a single tool like RASS may help streamline bedside assessment processes and reduce the need for repeated evaluations using different instruments.

However, whether RASS can fully replace SBS in mechanically ventilated children requires careful consideration. The high level of agreement observed in this study supports its potential as an alternative tool for sedation assessment in most clinical scenarios. Nevertheless, given the observed discrepancies in deep sedation and the fact that SBS was specifically developed and validated for pediatric ventilated patients (23, 26), complete substitution may not be appropriate in all situations. Clinical context, patient condition, and the need for precise assessment should guide tool selection. In addition, successful implementation of RASS in pediatric settings depends on standardized training and consistent application to ensure reliability (12, 25). Therefore, RASS may be best positioned as a practical alternative or complementary tool that can reduce nursing workload and improve workflow efficiency, rather than as a universal replacement for SBS.

## 4 Conclusion

In this retrospective PICU cohort, RASS demonstrated a strong positive association and good clinical agreement with SBS in mechanically ventilated children. The results support the use of RASS as an efficient sedation assessment option in settings where staff receive standardized training and where local protocols clearly define assessment procedures. At the same time, the observed discrepancies in the deepest sedation category indicate that scale selection should consider patient condition, clinical workflow, and the need for precise monitoring. Strengthening goal-directed sedation assessment may help reduce avoidable extremes of sedation and improve the quality of pediatric critical care.

## 5 Limitations

Several limitations should be acknowledged. First, this was a retrospective observational study based on routinely collected clinical data, and no formal *a priori* sample size calculation was performed. The study period was determined by the availability of complete electronic sedation assessment records. Second, although patient-level cluster-robust standard errors and GEE sensitivity analysis were used to address repeated measurements, residual within-patient correlation and unmeasured time-varying clinical factors may still have influenced the estimates. Future prospective studies should consider mixed-effects models, prespecified sampling strategies, or single-time-point validation designs to further confirm these findings.

Third, the nurse questionnaire lacked detailed methodological validation, including formal assessment of reliability, validity, and sampling strategy, which may limit the reproducibility and generalizability of the survey findings. Fourth, although this study adjusted for age, duration of mechanical ventilation, and PRISM-III disease severity in the generalized estimating equation model to account for key confounding factors, some important clinical factors—such as the use of vasoactive medications, the presence of neural developmental delay, and specific analgesic regimens—could not be fully incorporated into the analysis due to limited data availability in this retrospective cohort. Residual confounding effects resulting from these unmeasured variables could not be ruled out. Future multicenter prospective studies with standardized data collection, validated usability instruments, and more comprehensive adjustment for clinical confounders are warranted.

## Data Availability

The original contributions presented in the study are included in the article/Supplementary Material, further inquiries can be directed to the corresponding author.
