# Supplementary material for: Comparative evaluation of SBS and RASS for sedation assessment in mechanically ventilated children
Source: Front Pediatr. 2026 May 25;14:1829799. doi: 10.3389/fped.2026.1829799 (PMC13243422; doi:10.3389/fped.2026.1829799)

1. Sedation Scale Conversions: SBS and RASS：

| Sedation Scale Conversions | |  |
| --- | --- | --- |
| RASS | SBS |  |
| **+4 Combative** Combative, violent, immediate danger to staff | **+2 Agitated** May have difficulty breathing with ventilator Coughing spontaneously No external stimulus required to elicit response Spontaneously pays attention to care provider Unsafe (biting ETT, pulling at lines, cannot be left alone) Unable to console Increased movement (restless, squirming or thrashing side-to-side, kicking legs) |  |
| **+3 Very Agitated** Pulls to remove tubes or catheters; aggressive |  |  |
| **+2 Agitated** Frequent non-purposeful movement, fights ventilator | **+1 Restless and difficult to calm** Spontaneous effective breathing/Having difficulty breathing with ventilator Occasional spontaneous cough Responds to voice/No external stimulus is required to elicit response Drifts off/Spontaneously pays attention to care provider Intermittently unsafe Does not consistently calm despite 5 minute attempt/unable to console Increased movement (restless, squirming) |  |
| **+1 Restless** Anxious, apprehensive, movements not aggressive |  |  |
| **0 Alert and Calm** Spontaneously pays attention to caregiver | **0 Awake and able to calm** Spontaneous and effective breathing Coughs when repositioned/Occasional spontaneous cough Responds to voice/No external stimulus is required to elicit response Spontaneously pays attention to care provider Distresses with procedures Able to calm with comforting touch or voice when stimulus removed Occasional movement of extremities or shifting of position/increased movement (restless, squirming) |  |
| **-1 Drowsy** Not fully alert, but has sustained awakening to voice – eye opening and contact >10 seconds | **-1 Responsive to gentle touch or voice** Spontaneous but ineffective nonsupported breaths Coughs with suctioning/repositioning Responds to touch/voice Able to pay attention but drifts off after stimulation Distresses with procedures Able to calm with comforting touch or voice when stimulus removed Occasional movement of extremities or shifting of position |  |
| **-2 Light Sedation** Briefly awakens to voice – eyes open and contact <10 seconds |  |  |
|  |  |  |
| **-3 Moderate Sedation** Movement or eye opening to voice - no eye contact |  |  |
| **-4 Deep Sedation** No response to voice, but movement or eye opening to physical stimulation | **-2 Responsive to noxious stimuli** Spontaneous yet supported breathing Coughs with suctioning/repositioning Responds to noxious stimuli Unable to pay attention to care provider Will distress with a noxious procedure Does not move/occasional movement of extremities or shifting of position |  |
| **-5 Unarousable** No response to voice or physical stimulation | **-3 Unresponsive** No spontaneous respiratory effort No cough or coughs only with suctioning No response to noxious stimuli Unable to pay attention to care provider Does not distress with any procedure (including noxious) Does not move |  |

2.Agreement Matrix: RASS vs SBS Sedation Scales：


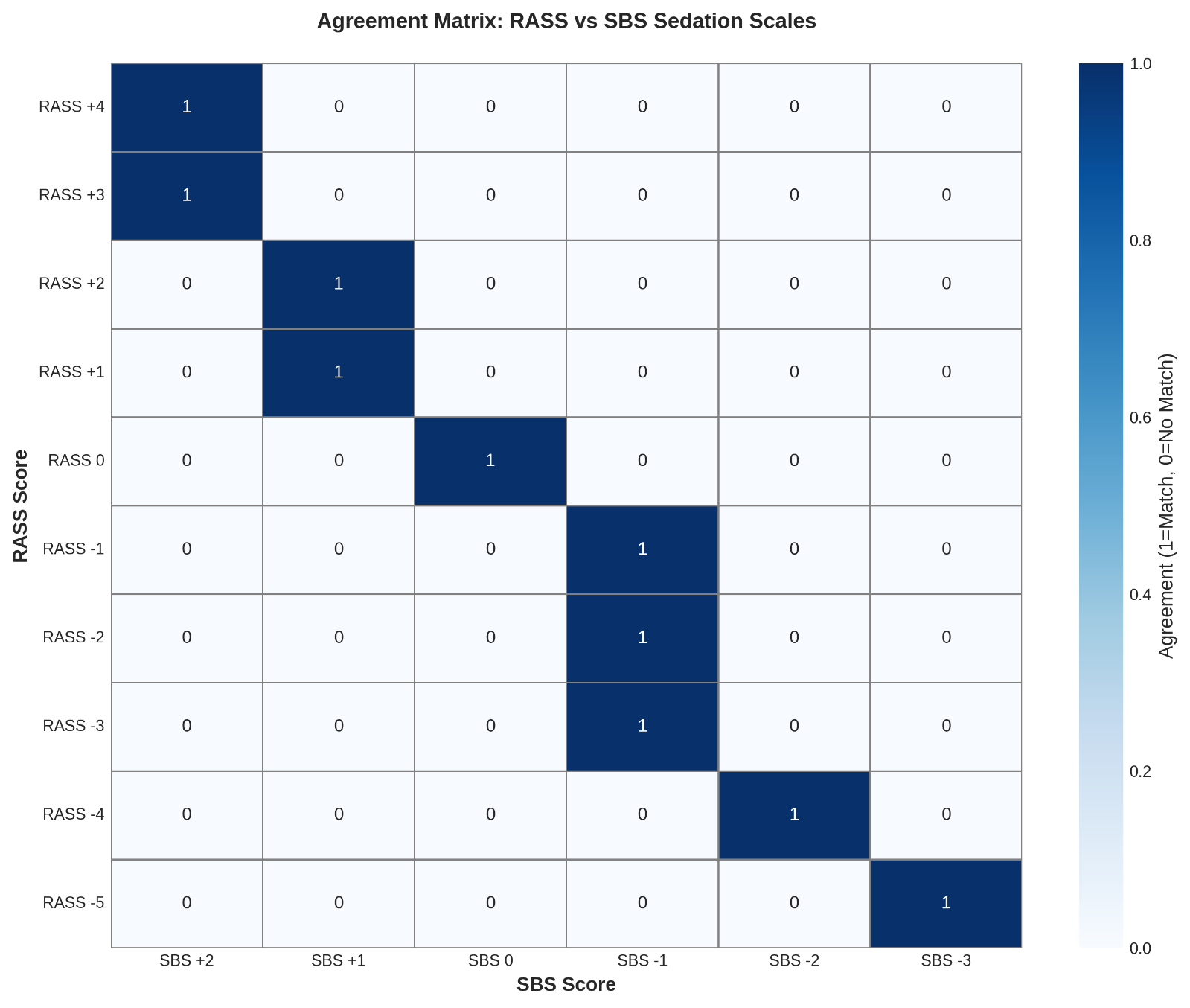


3.Absolute Frequency Heatmap: SBS vs RASS Sedation Scores：


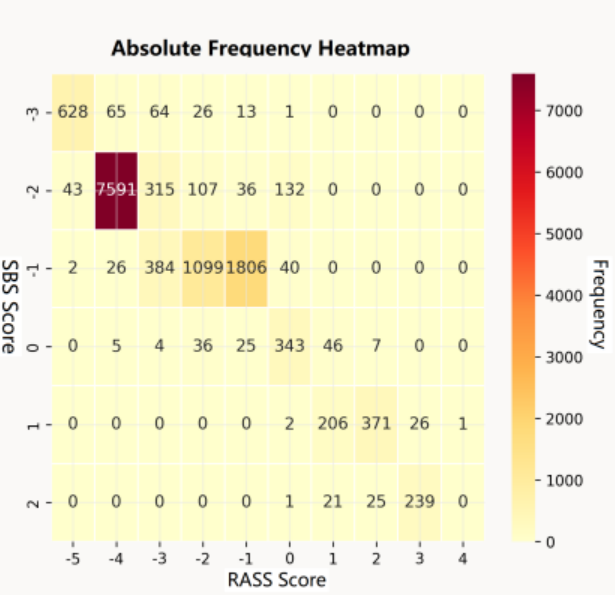

Supplement: Supplementary file 1 [file Supplementaryfile1.docx]
